# pH-Dependent Mechanisms of Influenza Infection Mediated by Hemagglutinin

**DOI:** 10.3389/fmolb.2021.777095

**Published:** 2021-12-17

**Authors:** Michael Caffrey, Arnon Lavie

**Affiliations:** Department of Biochemistry and Molecular Genetics, University of Illinois at Chicago, Chicago, IL, United States

**Keywords:** dynamics, hemagglutinin, histidine, influenza, structure, therapeutics, x-ray

## Abstract

Influenza hemagglutinin (HA) is a viral membrane bound protein that plays a critical role in the viral life cycle by mediating entry into target cells. HA exploits the lowering of the pH in the endosomal compartment to initiate a series of conformational changes that promote access of the viral genetic material to the cytoplasm, and hence viral replication. In this review we will first discuss what is known about the structural properties of HA as a function of pH. Next, we will discuss the dynamics and intermediate states of HA. We will then discuss the specific residues that are thought to be titrated by the change in pH and possible mechanisms for the pH triggered conformational changes. Finally, we will discuss small molecules that disrupt the pH trigger and thus serve as potential therapeutic strategies to prevent influenza infection.

## Introduction

It has long been appreciated that protein structures are adapted to the pH of their environment. For example, the proteins of extremophiles living at very low pH (e.g., pH 2) are stable at acidic pH and unstable/precipitated at neutral pH (Reed et al., 2013). Alternatively, proteins of organisms living at neutral pH are typically unstable/precipitated at low pH (Talley and Alexov, 2010). Moreover, the proteins of animals have adapted to the appropriate pH of their location (i.e., neutral pH for the extracellular fluids and cytoplasm, pH ∼5 for the endosomal and lysosomal spaces, and pH 2 for proteins of the stomach, Garcia-Moreno, 2009). Interestingly, in certain cases viral proteins have evolved to exploit the acidification of the endosome to activate their method of penetration into the cytoplasm of animal cells (Helenius, 2013). In this review we will discuss the pH related properties of influenza hemagglutinin (HA), a viral protein that has adapted to exploit the change from neutral pH (in the extracellular fluid) to pH ∼5 (in the endosome of target cells) for the purpose of allowing the viral genetic material access to the cytoplasm (White et al., 1982).

In the case of influenza A and B, the virus introduces its genetic material into the target cell by a series of well-orchestrated events mediated by HA, a membrane bound viral protein (Skehel and Wiley, 2000; Eckert and Kim, 2001; Luo, 2012). First, the virus binds to the target cell extracellular membrane (lung cells in humans) via an interaction between HA and its receptor, sialic acid bound to glycoproteins and glycolipids on the cell surface. Next, the virus enters the cell by endocytosis. Strikingly, the virus exploits the lowering of the pH as the endosome matures. The lower pH stimulates a series of conformational changes in HA that ultimately lead to the fusion of the viral membrane with the endosomal membrane, thereby allowing the introduction of the viral genetic information into the target cell cytoplasm. In what follows we discuss the implications of this change in pH to HA structure and dynamics. In addition, we discuss potential residues that serve as pH sensors and triggers, the mechanism of the triggering event, and potential strategies to disrupt the pH triggering event by therapeutic molecules.

## HA-Mediated Virus Entry

**FIGURE 1:**
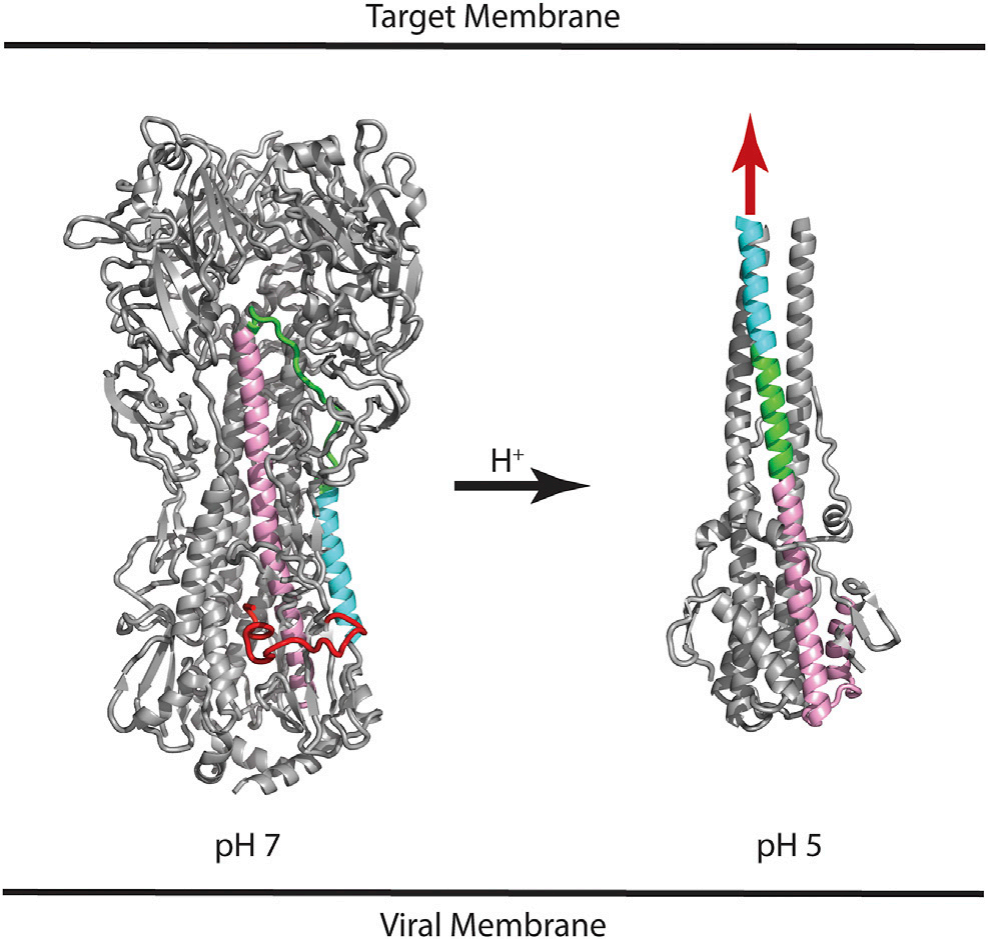
Structures of influenza HA at neutral and low pH. Based on the presence of the C-terminal transmembrane domain, the virus membrane is at the bottom of the panel. Based on the location of the receptor binding domain, the target membrane is located at the top of the panel. Coloring scheme: Fusion peptide (HA2 residues 1–21), red; N-helix (HA2 residues 37–57), cyan; Stem loop (HA2 residues 58–74), green; C-helix (HA2 residues 75–126), pink. In the case of the low pH structure, the HA1 subunit and fusion peptide of HA2 are missing from the construct; the purported location of the fusion peptide is shown as a red arrow. For simplicity, only one of the three subunits is colored. PDB files correspond to 2FK0 and 1HTM for the pH 7 and pH 5 structures, respectively.

HA is synthesized as a protomer of ∼520 residues (referred to as HA0), which undergoes posttranslational glycosylation and assembly into a trimer. Subsequently, HA0 is cleaved at position ∼345 to form the HA1 and HA2 subunits, which remain connected by non-covalent interactions and the presence of one intersubunit disulfide bond (Wilson et al., 1981; Gething et al., 1986). The HA1 subunit contains the receptor binding domain (RBD) and the HA2 subunit contains the fusion peptide that is thought to directly interact with the target membrane during the membrane fusion event. Much is known about the mechanism of HA-mediated entry from crystal structures of the HA extracellular domain, which include the neutral pH structure, the unprocessed HA0 structure, and the low pH structure (Skehel and Wiley, 2000). Figure 1 shows the structure of HA in the neutral pH conformation with the fusion peptide shown in red, the virus membrane depicted below (based on the presence of a C-terminal transmembrane domain, which is missing from the crystal construct), and the target membrane depicted at the “top” (based on the location of the RBD located at the “top” of the molecule). Following binding to the target membrane and uptake of the virus by endocytosis, HA is exposed to acidification of the endosome that ultimately results in a large change in conformation at pH ∼5 (Figure 1). The low pH structure shows that the stem loop, shown in green, undergoes a transition from a coil to a helix to form a long helix, comprised of the N-helix, stem loop, and parts of the C-helix, with the fusion peptide thrust toward the target membrane, sometimes referred to as a harpooning action (Eckert and Kim, 2001). Subsequently, additional changes in conformation are thought to occur that bring the virus and endosomal membranes in close contact, resulting in fusion of the membranes and introduction of the viral RNA into the target cell cytoplasm. However, there is one important caveat to this structure-based model: the low pH structure was generated from a highly manipulated construct, termed TBHA2, that is missing >50% of the HA residues (including the fusion peptide and transmembrane domains) due to the challenges of crystallization (Skehel and Wiley, 2000). Nonetheless, there are a large number of observations that support the proposed mechanism. First, the unprocessed form, HA0, does not allow membrane fusion and is noninfectious, presumably due to the absence of a “free” fusion peptide (Skehel and Wiley, 2000). Furthermore, influenza pathogenicity is correlated with the cleavability of HA0 (Steinhauer, 1999). Second, the acidification step is critical to HA function, as shown by studies with drugs neutralizing the endosome (e.g., ammonium chloride or chloroquine; Helenius, 2013). Third, *in vitro* studies show that the HA fusion peptide directly interacts with model membranes (Epand et al., 2001). Fourth, *in vitro* studies of the stem loop show that it undergoes a coil to helix transition at low pH (Carr et al., 1997). Finally, structures of other viral envelope proteins that mediate fusion (e.g., HIV gp41 and Ebola gp2), as well as cellular proteins that mediate fusion (e.g., SNARES), show a similar low energy final state with N-terminal domains in proximity with C-terminal domains, suggesting a conserved fusion mechanism (Harrison, 2008). Taken together, the importance of pH to HA function and the model for a pH induced conformational change are strongly supported.

## Intermediate States of HA

**FIGURE 2:**
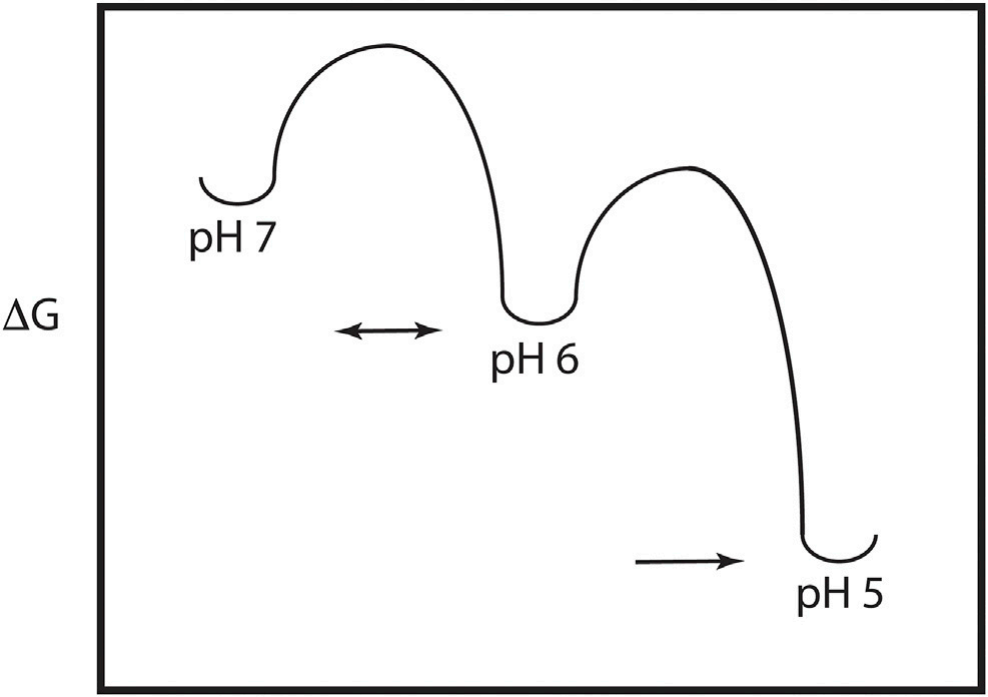
Energy plot of the pH trigger in HA. Note that there may be multiple intermediate states and that the transition to some of the intermediate states may be reversible.

As discussed above, the HA model requires significant pH induced changes in structure, which implies the presence of a metastable and dynamic/flexible state at neutral pH (Carr et al., 1997). Moreover, the acidification process in the endosome results in HA being exposed to a series of decreasing pH environments, potentially resulting in intermediate states at intermediate pH values. Consequently, one expects that the neutral pH structure is at a higher energy than the low pH structure, with intermediate energies in between as depicted in Figure 2. This model of HA intermediate states in dynamic equilibrium is supported by a large number of observations. First, as discussed above x-ray studies support a large conformational change from the neutral pH structure to the low pH structure (Skehel and Wiley, 2000; Eckert and Kim, 2001). Second, there are x-ray and EM structures of intermediates in reversible exchange (Xu and Wilson, 2011; Fontana et al., 2012; Antanasijevic et al., 2020a; Benton et al., 2020). Third, virological studies of influenza virus exposed to intermediate pH values imply reversible conformations (Leikina et al., 2002). Fourth, MS H/D exchange increases at intermediate pH suggesting increased exposure of exchangeable groups and dynamics/flexibility (Garcia et al., 2015). Fifth, the binding of an inhibitor peptide to a nonexposed region of HA suggests the presence of large breathing motions (Kingsley et al., 2017). Sixth, molecular dynamics studies of HA suggest the presence of intermediate conformations and larger flexibility at low pH (Lin et al., 2016; Burke et al., 2020). Finally, fluorescence studies of HA show the presence of multiple intermediate states in reversible exchange (Das et al., 2018). Consequently, the current model of HA-mediated entry requires multiple conformational states, within and between pH regimes. Moreover, many, but not all of the states, are in dynamic equilibrium determined in part by the height of the activation energy (i.e., HA-mediated entry is driven by kinetic and thermodynamic factors, Baker and Agard, 1994).

## Mechanism of the pH Trigger

The pH trigger is a critical feature found in influenza A and B HA. Consequently, there are two pertinent aspects to discussion of the pH trigger mechanism: identification of pH sensing residues and details of the pH-induced mechanism. With respect to the first aspect, one or more histidine residues would appear to be the most likely sensors based on the sidechain pKa value in the range of 5–7, the pH where the irreversible conformation change takes place (Kampmann et al., 2006; Zhou et al., 2014; Mair et al., 2014). However, it must be noted that aspartate and glutamate sidechains, which normally have pKa of ∼4, may exhibit significantly higher pKa in hydrophobic environments (Harms et al., 2009) and thus the acidic residues may also potentially participate in the pH trigger. Interestingly, HA-mediated membrane fusion occurs at different pH values for different HA subtypes in influenza A (Scholtissek, 1985; Puri et al., 1990; Galloway et al., 2013). For example, Galloway et al. (2013) have shown that the pH of fusion occurs over a range of 0.7 pH units for different subtypes, most likely due to subtle differences in the environment of the relevant histidine sidechains (and possibly aspartate and glutamate sidechains). Moreover, it appears that the modulation of the pH transition of HA is related to adaptation of influenza A subtypes to their host. (Di Lella et al., 2016; Gerlach et al., 2017; Yang et al., 2021).

**FIGURE 3:**
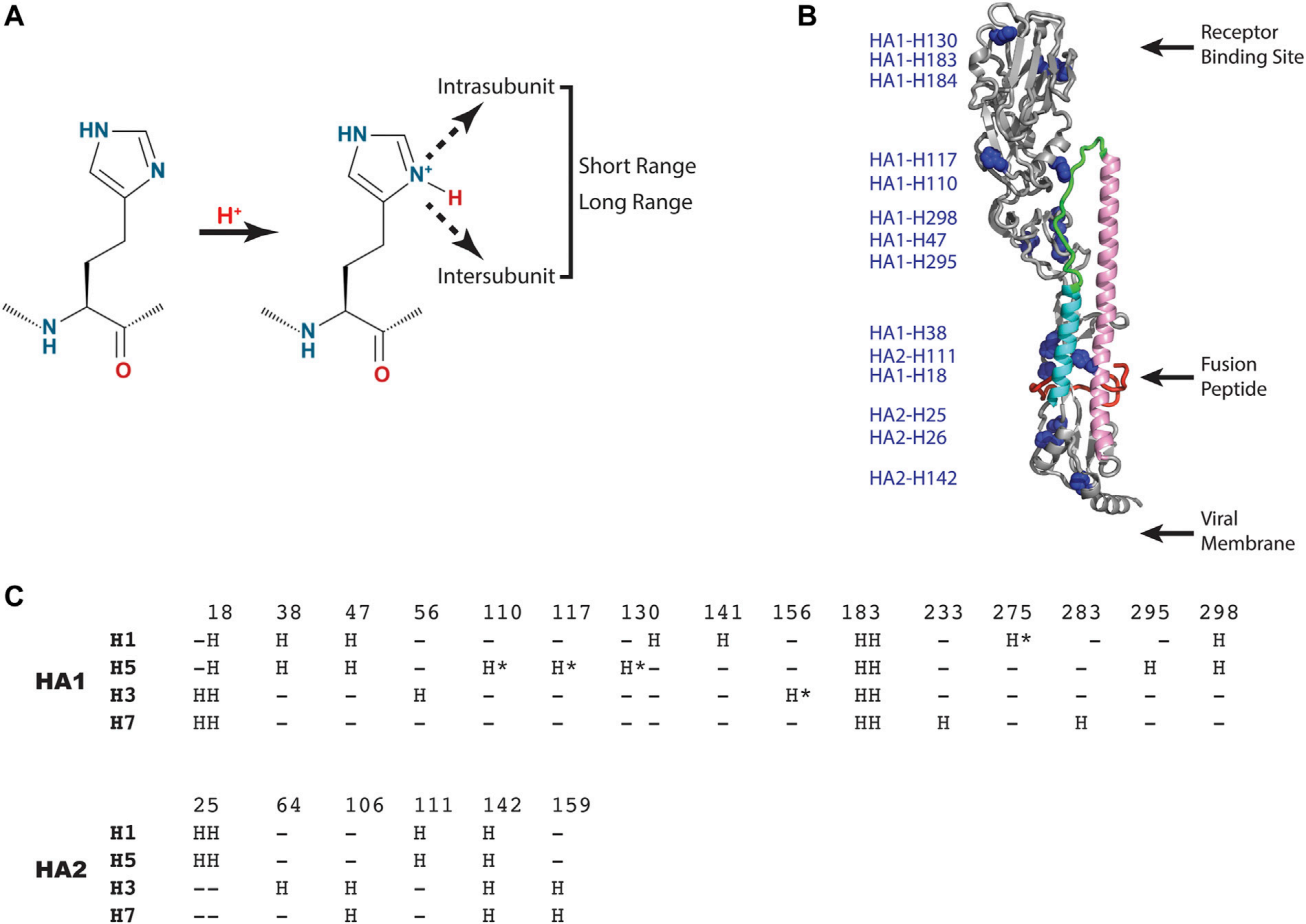
HA residues implicated in the pH trigger. **(A)** Schematic representation of the type of interactions that could occur upon protonation of select histidine sidechains. **(B)** Distribution of histidine residues observed in a representative HA (H5 HA; pdb = 2FK0). The coloring scheme of the backbone is that of Panel 1 with histidine sidechains shown as blue spheres. For simplicity, only one monomer of the HA trimer is shown. **(C)** Sequence alignment of consensus histidine residues found in representative Group 1 and 2 HA of influenza A using CLUSTAL O (1.2.1). Sequences: H1 HA (A/Puerto Rico/8/1934 H1N1); H5 HA [A/Viet Nam/1203/2004(H5N1)], H3 HA [A/Brisbane/10/2007(H3N2)] and H7 HA [A/Netherlands/219/2003(H7N7)]. The consensus histidines are found in >99% of the influenza A sequences deposited in the NCBI influenza virus data base and the nonconsensus histidines are denoted by asterisks.

As shown in Figure 3A, the histidine model predicts that one or more histidine sidechains become protonated, which results in a new positive charge, and that the resulting positive charge is sensed by short range interactions within or between subunits (HA1 or HA2) or propagated to long range interactions. Notably, the new interactions could be repulsive (e.g., positive-positive), attractive (e.g., positive-negative), or disruptive (e.g., introduction of a charge in a hydrophobic pocket). Analysis of a representative influenza A HA structure shown in Figure 3B reveals that histidine residues are distributed throughout the HA1 and HA2 subunits, in many cases as clusters of histidines, which could lead to repulsive forces upon protonation. Interestingly, one of these clusters (comprised of HA1-H18, HA1-H38 and HA2-H110) is in close proximity to the hydrophobic fusion peptide, which transitions to an exposed domain in the low pH structure (Figure 1). Moreover, another histidine cluster is found in close proximity to the stem loop (comprised of HA1-H47, HA1-110, HA1-117, HA1-295 and HA1-298), which transitions to a helix in the low pH structure (Figure 1). Since the pH trigger mechanism is highly conserved in all influenza HA, it is informative to examine the conservation of histidine residues in influenza A HA subtypes [influenza A subtypes were chosen because they have a higher degree of sequence identity with respect to influenza B HA, which exhibits ∼20% sequence identity to influenza A HA, despite a very high degree of structural homology (Wang et al., 2008)]. For this analysis we have chosen 4 diverse HA subtypes, 2 from Group 1 (H1 and H5 HA), and 2 from Group 2 (H3 and H7 HA) of influenza A. H1 and H3 are the primary subtypes circulating in humans and H5 and H7 HA are primarily found in birds but are highly pathogenic in humans (causing the “bird flu”) (Nobusawa et al., 1991; Webster et al., 1992; Hui and Zumla, 2015). As shown in Figure 3C, 4 consensus histidines are conserved in all 4 subtypes (HA1-H18, HA1-183, HA1-184, HA2-142), making these attractive candidates for the pH trigger. In the case of HA1-H18, it is in close proximity to the fusion peptide, and it has been implicated as a potential pH trigger by mutagenesis studies (Reed et al., 2009). On the other hand, it could be that the pH trigger function is conserved without conservation of discreet residues (e.g., by complex networks of ionizable groups). Finally, it is interesting to assess existing models for the pH trigger. Recently, we have proposed that HA1-H18 and HA1-H38 are implicated in the pH trigger in H5 HA, based on x-ray crystal structures of HA at neutral, intermediate and low pH (Antanasijevic et al., 2020a). Specifically, we observed that at intermediate pH values the HA1-H38 sidechain rotates away from the HA1-H18 sidechain, presumably due to cation-cation repulsion and subsequent stabilization by a newly formed electrostatic interaction with the carboxyl group of HA1-E24 (Antanasijevic et al., 2020a). Notably, HA1-H38 is in close proximity to the fusion peptide and thus the low pH conformation may result in increased solvent exposure and dynamics of the hydrophobic fusion peptide. In summary, pH triggering of HA is a highly conserved mechanism; however, the observed differences in the fusion pH of different subtypes, as well as the limited degree of conservation of histidine residues, suggests that pH mediated conformational changes of HA are most likely to be due to complex networks of conserved and non-conserved residues, with selection within each subtype.

## Exploitation of the pH Trigger

**FIGURE 4:**
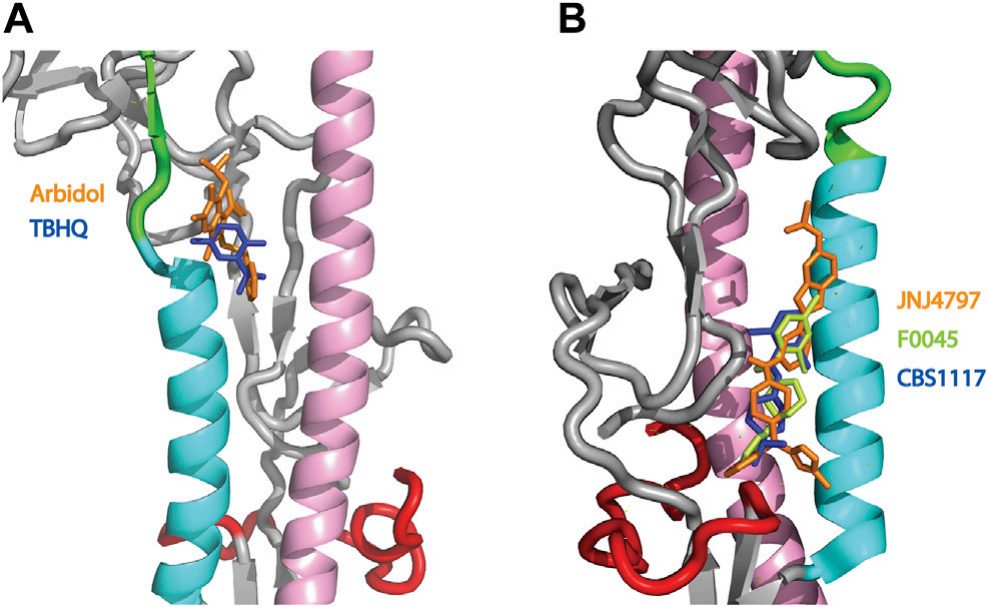
Structures of small molecule inhibitors thought to disrupt the pH trigger of HA. **(A)** Overlay of Group 2 inhibitors TBHQ (blue) and Arbidol (orange). PDB files correspond to 3EYM and 5T6N for the TBHQ and Arbidol complexes, respectively. **(B)** Overlay of Group 1 inhibitors JNJ4797 (orange), F0045 (lime) and CBS1117 (blue). PDB files correspond to 6FCG, 6WCR and 6VMZ for the JNJ4797, F0045 and CBS1117 complexes, respectively. In both panels, the coloring scheme of the backbone is that of Panel 1 **.**

HA plays a critical role in influenza infection and thus it is a potential target for therapeutic interventions (Lagoja and De Clercq, 2008). As discussed above, the neutral pH conformation of HA is considered to be metastable and acidification of the endosome results in a large conformation change to a more stable conformation with exposure of the HA fusion peptide (Skehel and Wiley, 2000; Eckert and Kim, 2001; Luo, 2012, Figure 1). To date there are 5 structures of HA (from influenza A) in complex with small molecules that inhibit the pH-mediated conformation change (Russell et al., 2008; Kadam and Wilson, 2017; van Dongen et al., 2019; Antanasijevic et al., 2020b; Yao et al., 2020). In the case of small molecule inhibitors TBHQ and Arbidol, they bind to the neutral conformation of Group 2 HA (e.g., H3 and H7 HA) in close proximity to the stem loop (Figure 4A) and they have been shown to disrupt the transition to the low pH form by stabilization of the neutral pH form (Russell et al., 2008; Antanasijevic et al., 2013; Kadam and Wilson, 2017). Interestingly, in Group 2 HA, the histidine cluster of HA1-H18 and HA1-H38 discussed above for Group 1 HA is missing (Figures 3B,C); however, the conserved histidine cluster of HA1-H17 and HA1-H18 found in Group 2 HA may participate in the pH transition (Figure 3C). Accordingly, TBHQ and Arbidol may function by stabilizing the stem loop domain through intrasubunit and intersubunit interactions, thereby overcoming the charge induced destabilization of another region. In the case of small molecule inhibitors JNJ4797, F0045 and CBS1117, they bind to the neutral conformation of Group 1 HA in close proximity to the fusion peptide and the N-helix (Figure 4B). As noted above there is a cluster of histidines nearby that may participate in the pH transition (HA1-H18, HA1-H38 and HA2-H111, Figure 3B). Hence JNJ4797, F0045 and CBS1117 may function by altering the pKa values of the nearby histidine (or acidic) sidechains and/or stabilizing the fusion peptide and N-helix interactions via hydrophobic and hydrophilic interactions between the inhibitor and HA. In summary, the best characterized small molecule fusion inhibitors bind to different sites and appear to function by different mechanisms of action in Group 1 and 2 HA. With respect to the protonation of HA histidines, the Group 2 HA inhibitors (TBHQ and Arbidol) do not interact directly, rather they act in a long-range manner. In contrast the Group 1 HA inhibitors (JNJ4797, F0045 and CBS1117) are in close proximity to a highly conserved histidine cluster and thus may exhibit direct and/or indirect effects.

## Concluding Remarks and Future Outlooks

HA is one of the best studied proteins with over 700 entries in the PDB and >14,000 entries in PubMed. Notably, the structures of HA at neutral pH and low pH were determined in 1968. However, despite being one of the best studied proteins, the precise mechanism of the pH trigger is still largely unresolved and most likely to involve numerous conserved and nonconserved residues. Additional studies of the intermediate states by CryoEM, fluorescence, mass spectrometry, and molecular dynamics will be critical to understanding the pathway toward the low pH state of HA. In addition, efforts to directly monitor the titration of individual histidines by NMR, which represents the only technique that can directly monitor histidine protonation (Hansen and Kay, 2014), should be pursued. Moreover, future efforts should include detailed analyses of HA mutants from diverse subtypes at intermediate pH. For example, mutagenesis studies of implicated histidines or nearby residues changed to alter the local environment will be highly informative, particularly when coupled to structural, biochemical and modeling studies. In summary, there is much to be learned about the pH-mediated HA and the potential to exploit the pH trigger for the development of novel influenza therapeutics, and thus this topic merits renewed efforts across multiple disciplines.
